# High-Glucose-Induced Metabolic and Epithelial Stress in Grass Carp Intestinal Epithelial Cells Associated with Methylation-Related Transcriptional Responses

**DOI:** 10.3390/ijms27135732

**Published:** 2026-06-25

**Authors:** Linjie Qian, Wenqiang Jiang, Yan Lin, Siyue Lu, Xianping Ge, Linghong Miao

**Affiliations:** 1 Wuxi Fisheries College, Nanjing Agricultural University, Wuxi 214081, China; qianlinjiejie@gmail.com; 2 Key Laboratory for Genetic Breeding of Aquatic Animals and Aquaculture Biology, Freshwater Fisheries Research Center, Chinese Academy of Fishery Sciences, Wuxi 214081, China; jiangwen-qiang@ffrc.cn (W.J.); liny@ffrc.cn (Y.L.); lusiyue@ffrc.cn (S.L.)

**Keywords:** high-glucose stress, 5-methylcytosine, intestinal epithelial cells, *dnmt3b-cdx1b-sglt1*, transcriptional response

## Abstract

High-glucose exposure impairs intestinal metabolic homeostasis and barrier integrity in fish, but the transcriptional responses associated with high-glucose adaptation in fish intestinal epithelial cells remain incompletely understood. This study investigated whether exogenous 5-methylcytosine (5MC) alleviates high-glucose-induced metabolic and epithelial stress in grass carp (*Ctenopharyngodon Idella*) intestinal epithelial cells and whether these responses are associated with changes in DNA methyltransferase 3 beta (*dnmt3b*) expression and Caudal type homeobox 1b (*cdx1b*)/Sodium-glucose cotransporter 1 (*sglt1*)-related transcriptional responses. As exploratory in silico information, molecular docking predicted candidate complex conformations of DNMT3B with CDX1B and SGLT1, with binding energies of −37.2 and −25.9 kcal/mol, respectively. Functionally, *dnmt3b* knockdown significantly reduced *dnmt3b*, Interleukin 6 (*il6*), and Nuclear factor kappa B (*nfκb*) expression, while increasing *cdx1b*, *sglt1*, Solute carrier family 2 member 3a (*slc2a3a*), 6-Phosphofructo-2-kinase/fructose-2,6-bisphosphatase 4a (*pfkfb4a*), and Amine oxidase copper containing 1 (*aoc*1) expression (*p* < 0.05). CDX2/CDX1B-like immunoreactive protein and SGLT1 protein levels were also increased after *dnmt3b* knockdown (*p* < 0.05). Under high-glucose stress, exogenous 5MC exerted concentration-dependent effects. Specifically, 6 mM 5MC significantly reduced residual extracellular glucose, lactate dehydrogenase and diamine oxidase activities, and malondialdehyde content, while increasing glutathione content, cell viability, and cell migration (*p* < 0.05). These effects remained detectable after replacement with high-glucose medium for an additional 12 h. By contrast, 24 mM 5MC markedly increased lactate dehydrogenase activity and reduced cell viability, suggesting potential cytotoxicity (*p* < 0.05). S-adenosylmethionine (SAM) levels were significantly lower in the NC and 6 mM groups than in the HG, 12 mM, and 24 mM groups, suggesting changes in SAM-related one-carbon metabolic status rather than direct evidence of altered DNA methylation (*p* < 0.05). Exogenous 5MC, particularly at 6 mM, alleviated high-glucose-induced metabolic and epithelial stress in grass carp intestinal epithelial cells. These effects were accompanied by changes in several glucose metabolism- and inflammation-related genes. However, the cellular uptake, metabolic fate, DNA incorporation, methylation consequences, and causal roles of these gene-expression changes remain to be further verified.

## 1. Introduction

Caudal type homeobox 1b (Cdx1b) is an important transcriptional regulator of intestinal epithelial specification and functional maturation in fish. In zebrafish, cdx1b is required for intestinal differentiation and acts as a functional equivalent of mammalian Cdx2 [1]. More recently, Jin et al. demonstrated that cdx1b protects intestinal cell fate by repressing signaling networks associated with liver specification during gut development [2]. In grass carp (*Ctenopharyngodon idella*), Cdx-family transcriptional regulation has also been linked to intestinal nutrient transport, as CDX2 was shown to directly regulate the intestinal oligopeptide transporter PepT1 and promote oligopeptide transport [3]. Glucose absorption in intestinal epithelial cells mainly depends on facilitative glucose transporters of the SLC2A/GLUT family and sodium-dependent glucose cotransporters of the SLC5A/SGLT family. Among them, SGLT1 is a key apical glucose transporter, and its molecular characterization in blunt snout bream (*Megalobrama amblycephala*) suggests an important role in intestinal glucose uptake and systemic glucose reabsorption [4]. In mammals, intestinal SGLT1 expression has also been implicated in the regulation of glycemia and body weight [5]. In addition, glucose transporters may participate in broader metabolic–transcriptional remodeling; for example, GLUT3-dependent glucose uptake controls Th17 cell responses through glycolytic–epigenetic reprogramming [6]. Together, these findings suggest that intestinal transcription factors and glucose transporters may cooperate in regulating nutrient absorption and metabolic adaptation.

Carbohydrate utilization remains a major nutritional challenge in aquaculture because many fish species exhibit limited tolerance to high carbohydrate intake. In grass carp, plasma glucose changes rapidly after oral starch administration, indicating that circulating glucose is tightly regulated in vivo [7]. At the cellular level, high-glucose exposure has been widely used as an in vitro stress model to investigate glucose overload-induced metabolic disturbance, oxidative stress, apoptosis, glycogen accumulation, and impaired protein synthesis in fish cells [8]. Increasing evidence further suggests that high-carbohydrate nutrition can be associated with changes in DNA methylation and metabolic gene regulation in fish. In grass carp, whole-genome DNA methylation analysis showed that a high-carbohydrate diet altered methylation patterns in metabolic pathways related to hyperglycemia and lipid deposition [9]. In yellow catfish (*Tachysurus fulvidraco*), parental nutritional programming also affected glucose metabolism-related gene expression and global DNA methylation in offspring larvae [10]. DNMT3B is a de novo DNA methyltransferase involved in the establishment of DNA methylation patterns and has increasingly been linked to metabolic stress and energy homeostasis. Brown fat-specific Dnmt3b deficiency was reported to affect thermogenesis, energy expenditure, adiposity, and insulin sensitivity in female mice [11], while DNMT3B was shown to aggravate renal fibrosis in diabetic kidney disease through activation of the Wnt/β-catenin pathway under high-glucose-related pathological conditions [12]. However, whether *dnmt3b* participates in intestinal glucose transporter-related transcriptional responses in fish remains unclear.

5-Methylcytosine (5MC) is the predominant methylated cytosine base in DNA and represents a core molecular mark of DNA methylation [13,14]. However, 5MC itself is a modified DNA base rather than a canonical metabolically active methyl donor such as S-adenosylmethionine (SAM). DNA methyltransferases catalyze cytosine methylation using S-adenosylmethionine as the methyl donor, rather than using 5MC as a direct methyl source [15]. Nutritional status and methyl-donor availability can influence one-carbon metabolism, DNA methylation, and metabolic regulation [16], but whether exogenous 5MC affects high-glucose-induced injury and glucose metabolism-related responses in fish intestinal epithelial cells remains largely unexplored. Moreover, because DNA methylation is dynamic and context-dependent [17,18,19], direct methylation and functional validation are required before establishing a causal epigenetic mechanism. Based on this background, the present study used grass carp intestinal epithelial cells to investigate whether exogenous 5MC supplementation alleviates high-glucose-induced metabolic disturbance and epithelial injury. We further examined whether these effects are associated with changes in *dnmt3b* expression and transcriptional responses of *cdx1b*, *sglt1*, and other glucose metabolism-related genes. Considering the current lack of direct DNA methylation evidence and complete functional validation, the *dnmt3b–cdx1b–sglt1* pathway is interpreted here as a transcriptionally supported candidate regulatory route rather than a definitively proven DNA methylation-dependent mechanism.

## 2. Results

### 2.1. Molecular Docking Results of DNMT3B with CDX1B and SGLT1

**Figure 1:**
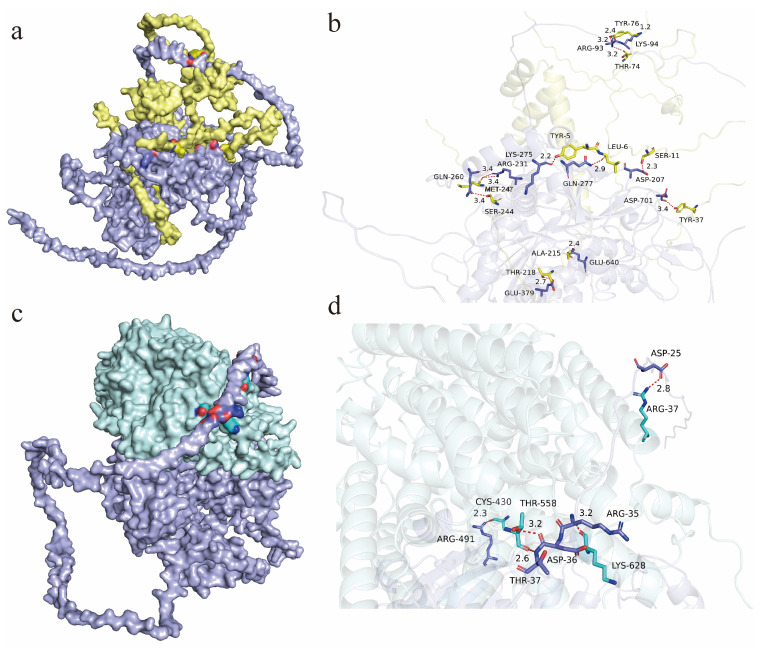
Exploratory molecular docking models of DNA methyltransferase 3 beta (DNMT3B) with Caudal type homeobox 1b (CDX1B) and Sodium-glucose cotransporter 1 (SGLT1). Note: (**a**,**b**) Molecular docking model of DNMT3B with CDX1B. (**a**) Overall conformation of the top-ranked candidate complex. (**b**) Enlarged view of the binding interface, where red dashed lines indicate hydrogen bond- or salt bridge-like interactions. The yellow region represents CDX1B, and the purple region represents DNMT3B. The predicted binding energy of this complex was −37.2 kcal/mol. (**c**,**d**) Molecular docking model of DNMT3B with SGLT1. (**c**) Overall conformation of the top-ranked candidate complex. (**d**) Enlarged view of the binding interface, where red dashed lines indicate hydrogen bond- or salt bridge-like interactions. The green region represents SGLT1, and the purple region represents DNMT3B. The predicted binding energy of this complex was −25.9 kcal/mol. These models are presented as in silico structural predictions and should not be interpreted as evidence of physiological protein–protein interaction or functional regulation.

As shown in Figure 1, the docking analysis revealed that both DNMT3B–CDX1B and DNMT3B–SGLT1 were predicted to form candidate complex conformations with favorable docking scores. Among them, the top-ranked DNMT3B–CDX1B complex exhibited a predicted binding energy of −37.2 kcal/mol (Figure 1a,b), whereas the predicted binding energy of the DNMT3B–SGLT1 complex was −25.9 kcal/mol (Figure 1c,d).

Further magnified views of the interaction interfaces showed that the major binding interface of DNMT3B–CDX1B involved residues MET-247, GLN-260, ARG-231, SER-244, LYS-275, and GLN-277, together with opposing interface residues including ASP-207, ASP-701, SER-11, and TYR-37. In comparison, the DNMT3B–SGLT1 complex also displayed a local interface maintained by multiple hydrogen bond- and salt bridge-like interactions, with candidate key residues including ASP-25, ASP-36, THR-37, ARG-35, ARG-37, ARG-491, CYS-430, THR-558, and LYS-628.

### 2.2. Expression of Metabolism- and Inflammation-Related Genes in Intestinal Epithelial Cells After dnmt3b Knockdown

**Figure 2:**
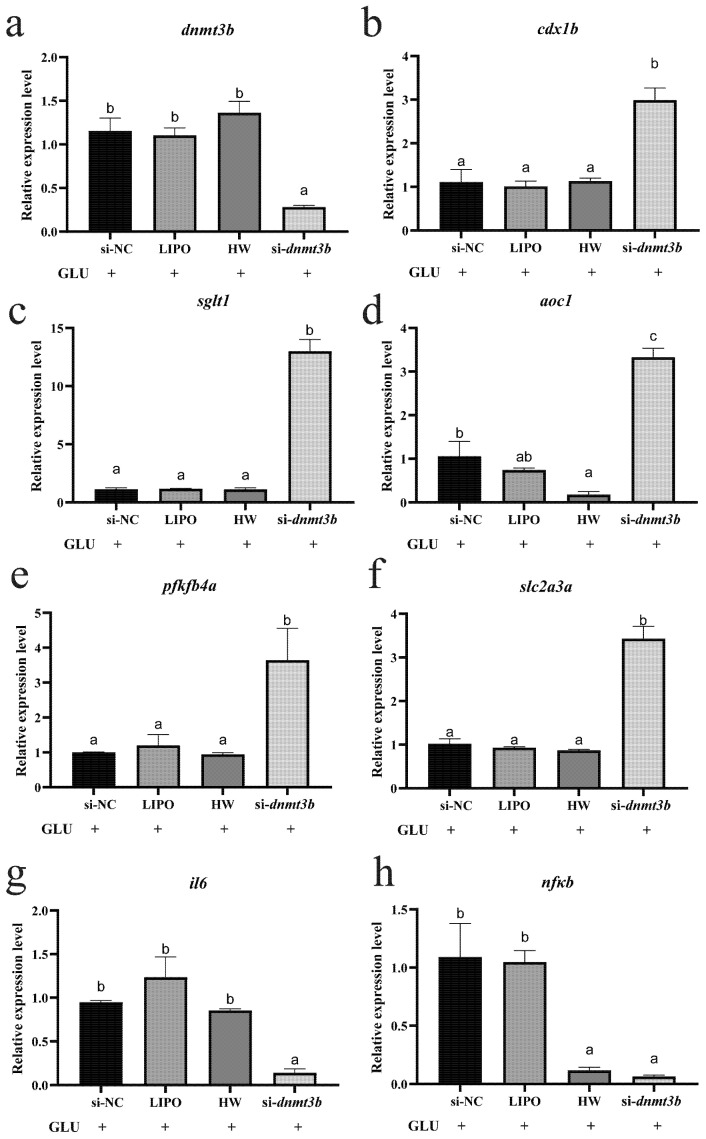
Relative mRNA expression levels of metabolism- and inflammation-related genes in grass carp intestinal epithelial cells after *dnmt3b* knockdown. (**a**) The relative expression of DNA methyltransferase 3 beta *(dnmt3b*); (**b**) The relative expression of Caudal type homeobox 1b (*cdx1b*); (**c**) The relative expression of Solute carrier family 5 member 1 (*sglt*1); (**d**) The relative expression of Amine Oxidase Copper Containing 1 (*aoc*1); (**e**) The relative expression of 6-Phosphofructo-2-kinase/fructose-2,6-bisphosphatase 4a (*pfkfb4a*); (**f**) The relative expression of Solute carrier family 2 member 3a (*slc2a3a*); (**g**) The relative expression of Interleukin 6 (*il*6) and (**h**) The relative expression of Nuclear factor kappa B (*nfκb*). The different lowercase letters in the figure indicate significant differences among si-NC; LIPO; HW and si-*dnmt3b* groups (Tukey, *p* < 0.05). + indicates the addition of glucose.

As shown in Figure 2, the expression levels of *dnmt3b* and Interleukin-6 (*il6*) in intestinal epithelial cells were significantly lower in the si-*dnmt3b* group than in the other three groups (*p* < 0.05). In addition, the expression levels of *cdx1b*, *sglt1*, Amine oxidase copper-containing 1 (*aoc1*), Phosphofructo-2-kinase/fructose-2,6-biphosphatase 4A (*pfkfb4a*), and *slc2a3a* were highest in the si-*dnmt3b* group (*p* < 0.05). Moreover, compared with the si-NC and LIPO groups, the expression level of Nuclear factor kappa-B (*nfκb*) was significantly reduced in both the HW and si-*dnmt3b* groups (*p* < 0.05).

### 2.3. Protein Levels of DNMT3B, CDX2/CDX1B-like and SGLT1 in Intestinal Epithelial Cells After dnmt3b Knockdown

**Figure 3:**
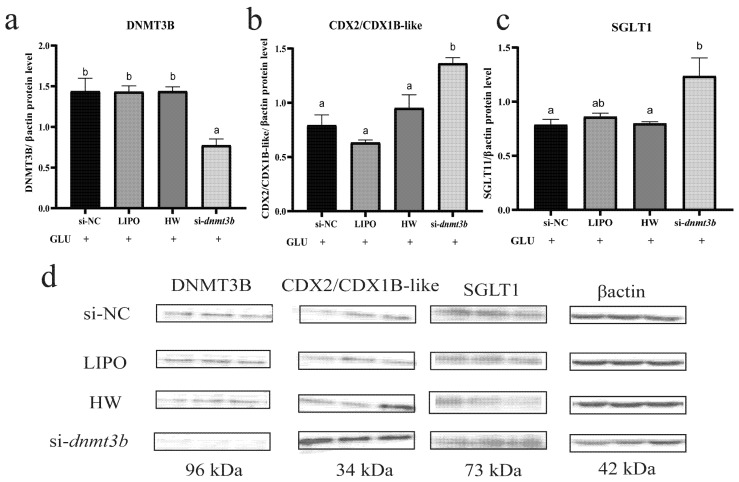
Protein levels of DNMT3B, CDX2/CDX1B-like immunoreactive protein, and SGLT1 in grass carp intestinal epithelial cells after *dnmt3b* knockdown. Note: CDX2/CDX1B-like in figure was CDX2/CDX1B-like immunoreactive protein. (**a**) The protein level of DNMT3B/βactin; (**b**) The protein level of CDX2/CDX1B-like/βactin; (**c**) The protein level of SGLT1/βactin. (**d**) Each group of WB protein images and their molecular weights. The different lowercase letters in the figure indicate significant differences among si-NC; LIPO; HW and si-*dnmt3b* groups (Tukey, *p* < 0.05). + indicates the addition of glucose.

As shown in Figure 3, the protein level of DNMT3B in intestinal epithelial cells was significantly lower in the si-*dnmt3b* group than in the si-NC, LIPO, and HW groups (*p* < 0.05). In contrast, the protein levels of CDX2/CDX1B-like immunoreactive protein and SGLT1 were significantly increased in the si-*dnmt3b* group (*p* < 0.05).

### 2.4. Effects of 5MC on High-Glucose-Induced Metabolic and Epithelial Stress in Intestinal Epithelial Cells

#### 2.4.1. Effects of 5MC Supplementation on Cell Survival Rate Under High-Glucose Stress

**Figure 4:**
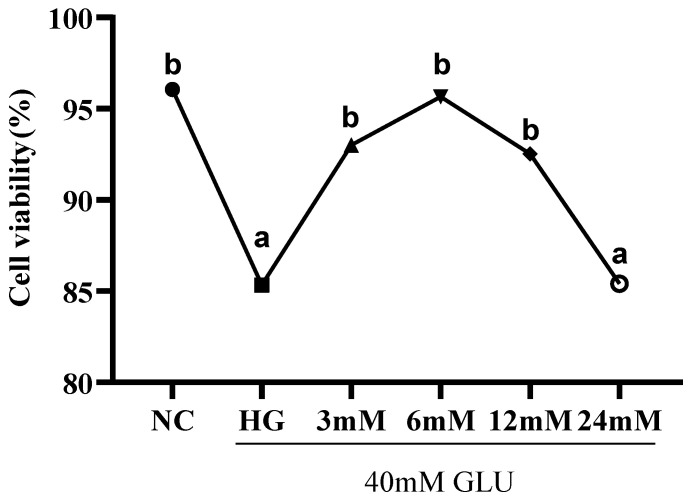
Effects of different 5MC concentrations on the viability of grass carp intestinal epithelial cells after 48 h of high-glucose exposure. The different lowercase letters in the figure indicate significant differences among NC; HG; 3–24 mM groups (Tukey, *p* < 0.05).

As shown in Figure 4, compared with the NC group, the cell survival rate in the HG and 24 mM group was significantly lower (*p* < 0.05). Among the 5MC-treatment groups, cell viability in the HG and 24 mM groups was significantly lower. The 3, 6, and 12 mM groups were significantly higher than the HG group (*p* < 0.05), and the 6 mM group had the highest rate.

#### 2.4.2. Effects of 5MC Supplementation on SAM Content Under High-Glucose Stress

**Figure 5:**
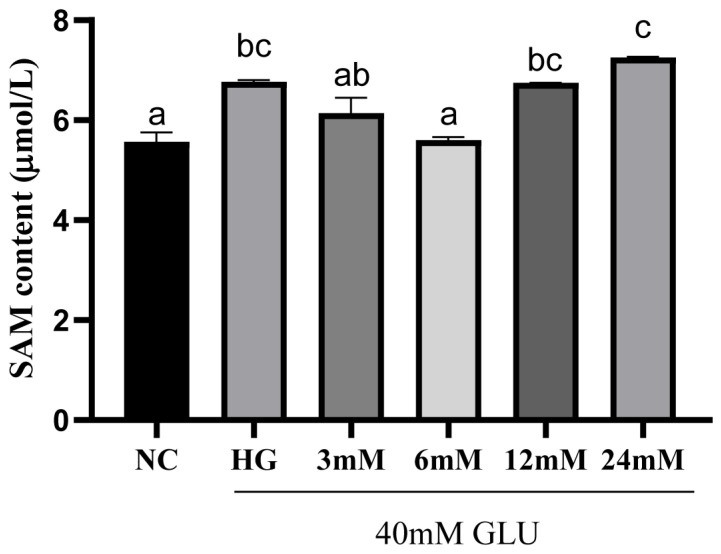
Effects of different concentrations of 5MC on SAM content of grass carp intestinal epithelial cells under high-glucose stress after 48 h of treatment. The different lowercase letters in the figure indicate significant differences among NC; HG; 3–24 mM groups (Tukey, *p* < 0.05).

As shown in Figure 5, compared with the NC and 6 mM groups, intracellular SAM content was significantly higher in the HG, 12 mM, and 24 mM groups than in the NC and 6 mM groups (*p* < 0.05).

#### 2.4.3. Biochemical Parameters in the Culture Supernatant After 48 h of 5MC Treatment

**Figure 6:**
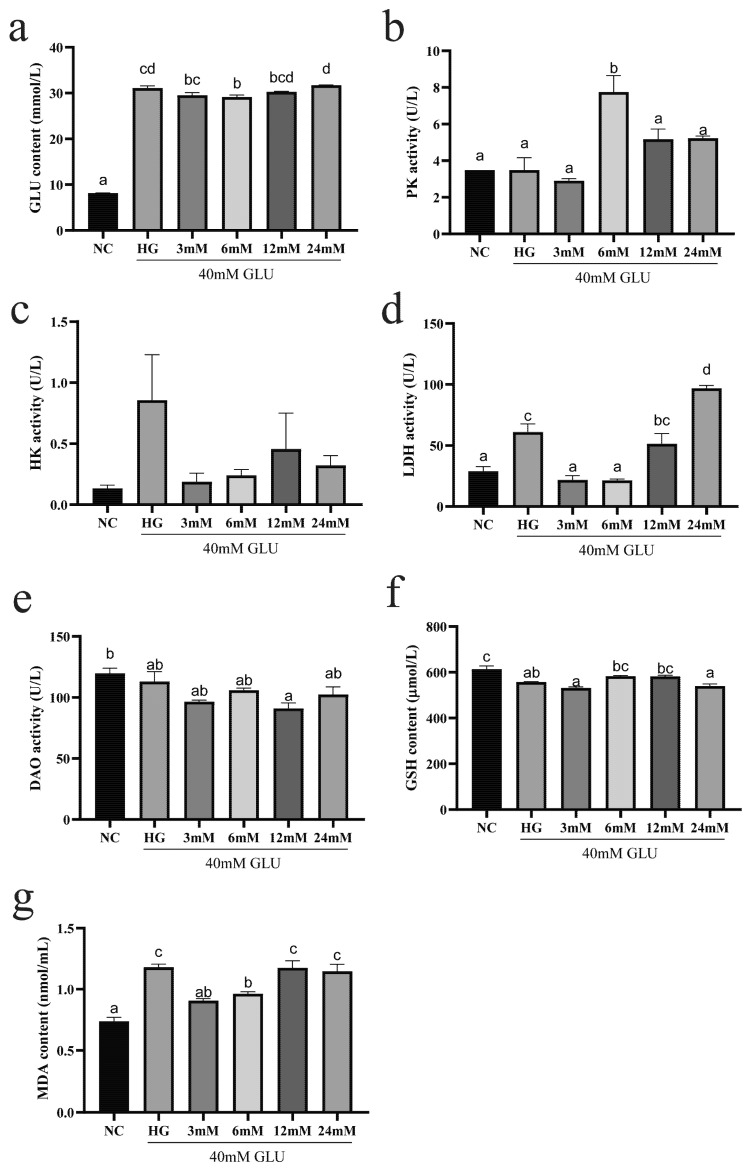
Biochemical results of culture medium supernatant 48 h after 5-Methylcytosine supplementation. (**a**) The content of glucose (GLU); (**b**) The activity of pyruvate kinase (PK); (**c**) The activity of hexokinase (HK); (**d**) The activity of lactate dehydrogenase (LDH); (**e**) The activity of diamine oxidase (DAO); (**f**) The content of glutathione (GSH); (**g**) The content of malondialdehyde (MDA). The different lowercase letters in the figure indicate significant differences among NC; HG; 3–24 mM groups (Tukey, *p* < 0.05).

As shown in Figure 6, compared with the NC group, the HG group exhibited significantly increased Glucose (GLU) and Malondialdehyde (MDA) contents as well as Lactate Dehydrogenase (LDH) activity, whereas Glutathione (GSH) content was significantly decreased (*p* < 0.05). Compared with the HG group, supplementation with 6 mM 5MC significantly reduced GLU and MDA contents and LDH activity in the culture supernatant (*p* < 0.05). The 6 mM group showed the highest Pyruvate Kinase (PK) activity among all groups (*p* < 0.05), whereas the 24 mM group exhibited significantly higher LDH activity than all other groups (*p* < 0.05). Diamine Oxidase (DAO) activity in the 12 mM group was significantly lower than that in the NC group (*p* < 0.05). In addition, compared with the NC, 6 mM, and 12 mM groups, GSH content was significantly decreased in the 3 mM and 24 mM groups (*p* < 0.05). No significant difference in Hexokinase (HK) activity was observed among the groups (*p* > 0.05).

#### 2.4.4. Effects of 5MC Supplementation on mRNA Expression Levels of Glucose Metabolism- and Inflammation-Related Genes

**Figure 7:**
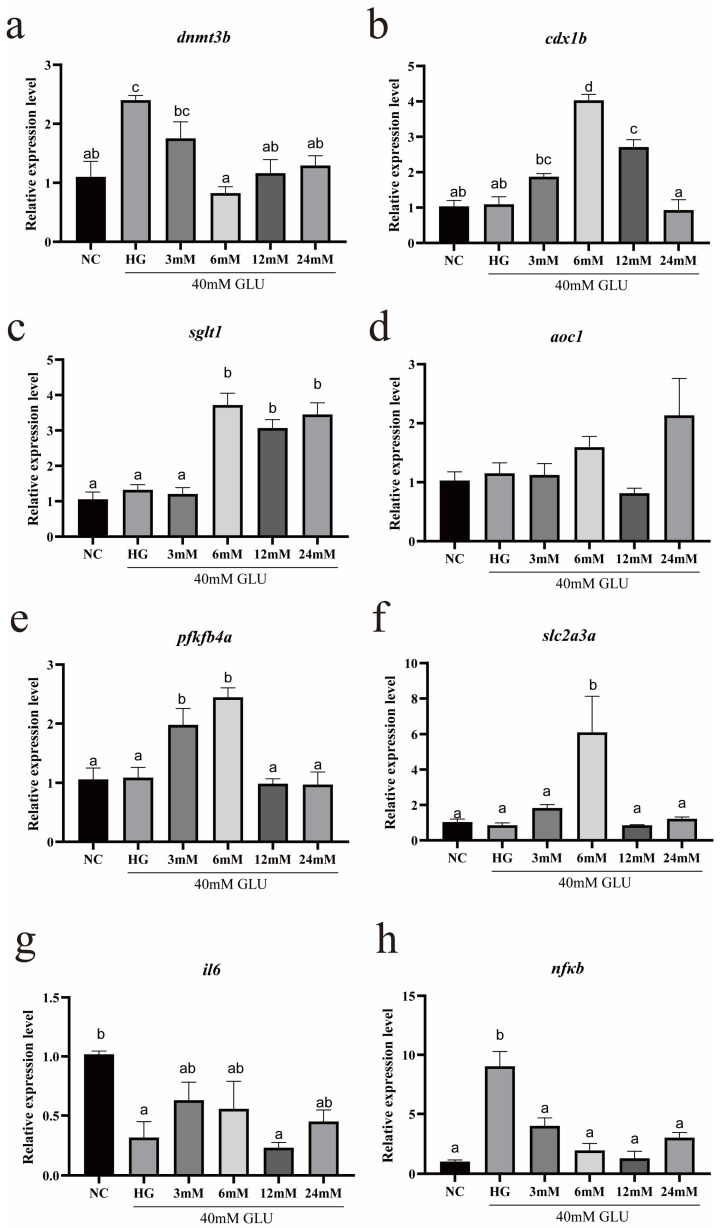
Effect of 5-Methylcytosine supplementation on the expression levels of related genes in intestinal cells. (**a**) The relative expression of *dnmt3b*; (**b**) The relative expression of *cdx1b*; (**c**) The relative expression of *sglt1*; (**d**) The relative expression of *aoc1*; (**e**) The relative expression of *pfkfb4a*; (**f**) The relative expression of *slc2a3a*; (**g**) The relative expression of *il6* and (**h**) The relative expression of *nfκb*. The different lowercase letters in the figure indicate significant differences among NC; HG; 3–24 mM groups (Tukey, *p* < 0.05).

As shown in Figure 7, compared with the HG group, the expression levels of *dnmt3b* and *nfκb* were significantly reduced in the 6 mM, 12 mM, and 24 mM groups (*p* < 0.05). The expression levels of *cdx1b* and *slc2a3a* were significantly higher in the 6 mM group than in all other groups (*p* < 0.05). Compared with the NC and HG groups, the expression levels of *sglt1* and *pfkfb4a* were significantly increased in the 6 mM group (*p* < 0.05). Similarly, the expression of *sglt1* in the 12 mM and 24 mM groups was also significantly higher than that in the NC and HG groups (*p* < 0.05). Compared with the NC group, the expression of *il6* was significantly lower in the HG and 12 mM groups (*p* < 0.05), whereas no significant differences were observed among the other groups (*p* > 0.05). In addition, *aoc1* expression did not differ significantly among groups (*p* > 0.05).

#### 2.4.5. Effects of 5MC Supplementation on Protein Levels in Intestinal Epithelial Cells

**Figure 8:**
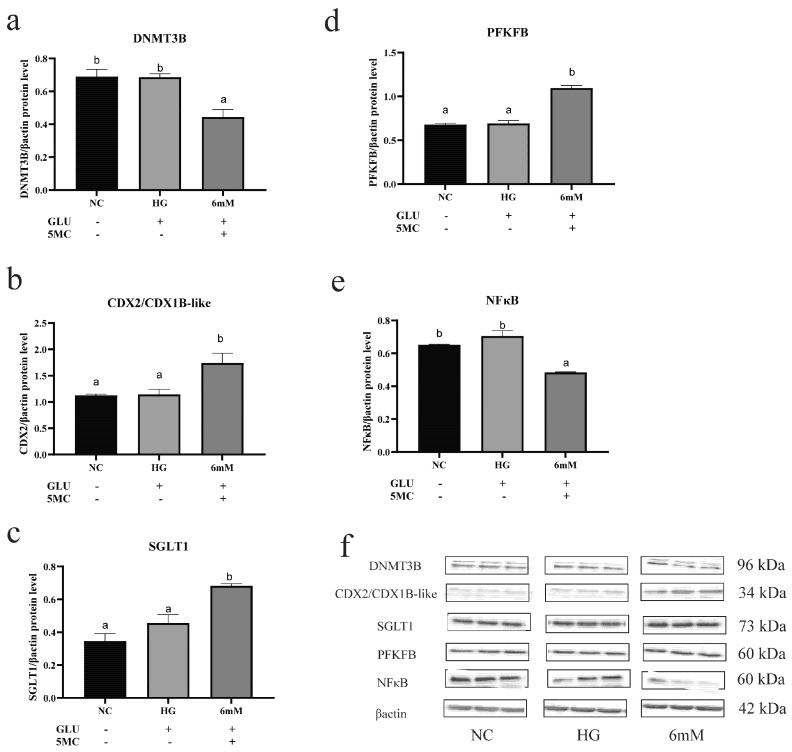
Effects of 5MC supplementation on protein levels of DNMT3B, CDX2/CDX1B-like immunoreactive protein, SGLT1, 6-Phosphofructo-2-kinase/Fructose-2,6-bisphosphatase (PFKFB), and Nuclear factor kappa B (NFκB) in intestinal epithelial cells. The protein level of (**a**) DNMT3B/βactin; (**b**) CDX2/CDX1B-like/βactin; (**c**) SGLT1/βactin; (**d**) PFKFB/βactin and (**e**) NFκB/βactin. (**f**) Each group of WB protein images and their molecular weights. The different lowercase letters in the figure indicate significant differences among NC; HG and 6 mM groups (Tukey, *p* < 0.05). + indicates addition, - indicates non-addition.

As shown in Figure 8, compared with the NC and HG groups, the 6 mM group exhibited significantly lower protein levels of DNMT3B and NFκB, whereas the protein levels of CDX2/CDX1B-like immunoreactive protein, SGLT1, and PFKFB were significantly increased (*p* < 0.05).

#### 2.4.6. Biochemical Parameters in the Culture Supernatant After 48 h of 5MC Treatment Followed by an Additional 12 h of Culture in High-Glucose Medium

**Figure 9:**
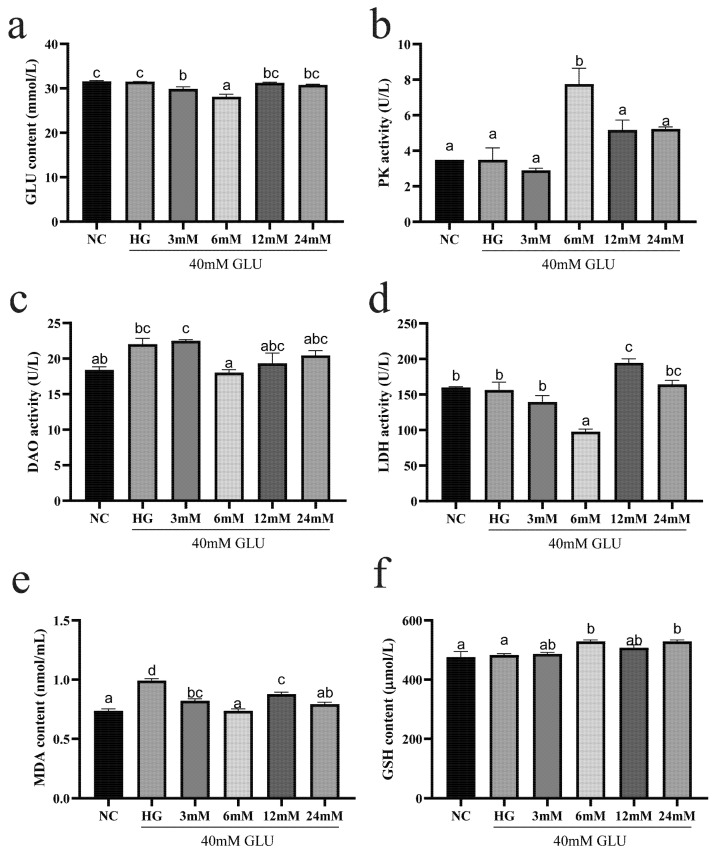
Biochemical results of the supernatant of the medium after 48 h of 5MC treatment followed by replacement with high-glucose medium for 12 h. (**a**) The content of GLU; (**b**) The activity of PK; (**c**) The activity of DAO; (**d**) The activity of LDH; (**e**) The content of MDA and (**f**) The content of GSH. The different lowercase letters in the figure indicate significant differences among NC; HG; 3–24 mM groups (Tukey, *p* < 0.05).

As shown in Figure 9, the 6 mM group exhibited significantly lower GLU content and LDH activity than all other groups (*p* < 0.05). Compared with the 3 mM group, PK activity was significantly reduced in the 6–24 mM groups (*p* < 0.05). In addition, compared with the HG and 3 mM groups, the 6 mM group showed significantly lower DAO activity and MDA content (*p* < 0.05). Compared with the NC and HG groups, GSH content was significantly increased in the 6 mM and 24 mM groups (*p* < 0.05).

### 2.5. Effects of 5MC on the Wound Healing Rate of Intestinal Epithelial Cells

**Figure 10:**
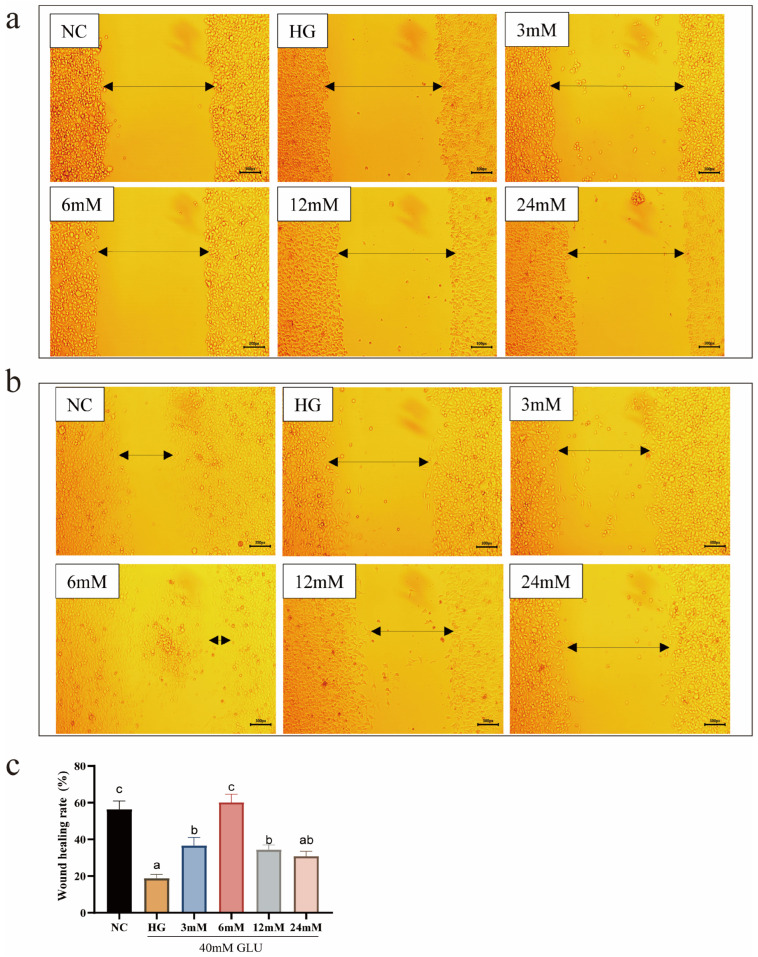
Effect of 5-Methylcytosine supplementation on intestinal cell migration. Note: (**a**) Cell scratch in each group at 0 h, where H represents wound width. (**b**) Cell scratch in each group at 24 h. (**c**) Cell migration rate, n = 9. The different lowercase letters in the figure indicate significant differences among NC; HG; 3–24 mM groups (Tukey, *p* < 0.05).

As shown in Figure 10, compared with the HG group, the cell migration rate significantly increased in the NC, 3 mM, 6 mM, and 12 mM groups (*p* < 0.05).

## 3. Discussion

### 3.1. Exploratory Molecular Docking Analysis DNMT3B with CDX1B and SGLT1

The molecular docking analysis showed that the predicted binding energy of the DNMT3B–CDX1B complex was −37.2 kcal/mol, whereas that of the DNMT3B–SGLT1 complex was −25.9 kcal/mol, suggesting higher predicted structural complementarity between DNMT3B and CDX1B. However, these docking results should be interpreted only as exploratory in silico predictions.

Previous studies have shown that CDX-family transcription factors are involved in intestinal epithelial identity and nutrient transporter regulation. DNA methylation status can influence CDX2 binding preference and genomic occupancy in a context-dependent manner [20], and CDX1 expression can be affected by promoter methylation [21]. In addition, the intestinal SGLT1 promoter contains binding sites for several transcription factors, including CDX2 [22]. These findings provide background support for a possible association between CDX-family transcription factors and SGLT1-related transcriptional regulation, but they do not prove that DNMT3B directly regulates CDX1B or SGLT1 in grass carp intestinal epithelial cells.

### 3.2. Effects of dnmt3b Knockdown on Glucose Metabolism-Related Transcriptional Responses in Grass Carp Intestinal Epithelial Cells

In mammals, three major DNA methyltransferases have been identified, namely DNMT1, DNMT3A, and DNMT3B. DNMT1 is primarily responsible for maintenance methylation, whereas DNMT3A and DNMT3B mainly mediate de novo DNA methylation. Increasing evidence indicates that DNMT3B is highly responsive to metabolic stress. In diabetic nephropathy models, DNMT3B expression is markedly induced in renal tubular epithelial cells under high-glucose conditions, and treatment with the methyltransferase inhibitor 5-azacytidine can reverse hyperglycemia-induced activation of the Wnt signaling pathway [12]. In brown adipose tissue, tissue-specific deletion of DNMT3B has been shown to promote thermogenesis, increase energy expenditure, reduce lipid accumulation, and improve insulin sensitivity in female mice [11]. Together, these findings suggest that DNMT3B may be involved in metabolic stress-related responses in some biological contexts.

In the present study, *dnmt3b* knockdown significantly increased the expression of *cdx1b* and *sglt1* in grass carp intestinal epithelial cells, suggesting that suppression of *dnmt3b* was associated with increased expression of several glucose transport-related genes. Consistent with this transcriptional response, the protein levels of CDX2/CDX1B-like immunoreactive protein and SGLT1 were also increased after *dnmt3b* knockdown, providing additional protein-level support for the involvement of a CDX-related transcription factor and SGLT1 in this response. This interpretation is supported by previous promoter analyses showing that CDX2/CDX1B-like can directly regulate SGLT1 promoter activity and cooperate with other transcription factors to determine intestinal SGLT1 expression [23]. Given the functional conservation within the CDX family, our results suggest a possible association between *cdx1b* expression and *sglt1*-related transcriptional responses in fish intestinal epithelial cells. This result is also broadly consistent with the docking prediction described above, in which DNMT3B showed a stronger predicted structural compatibility with CDX2/CDX1B-like than with SGLT1. However, because molecular docking alone cannot demonstrate physiological interaction, the docking data should be considered only as exploratory structural information rather than direct evidence of functional binding. In addition to *sglt1*, the upregulation of *slc2a3a* upregulation further supports a glucose transport-related transcriptional response. As a high-affinity facilitative glucose transporter, SLC2A3 is generally associated with metabolically active cells and tissues. Previous studies have shown that DNMT1-driven methylation can promote esophageal squamous cell carcinoma progression by modulating SLC2A3 transcription and glycolysis [24]. Moreover, Lin et al. reported that hypoxia enhances Methyltransferase-like 3 (METTL3)-mediated SLC2A3 m^6^A modification, thereby promoting glycolysis and osteogenesis in bone marrow mesenchymal stem cells [25]. Although these studies involve distinct biological contexts, they collectively highlight the importance of SLC2A3 in metabolic reprogramming. In our study, the concurrent upregulation of *slc2a3a* and *pfkfb4a* after *dnmt3b* knockdown suggests coordinated transcriptional changes in glucose transporter- and glycolysis-related genes. Therefore, these findings provide transcriptional and partial protein-level evidence for DNMT3B-associated *cdx1b/sglt1*-related responses, although direct functional and methylation analyses are still needed to clarify their biological significance under high-glucose stress.

PFKFB family members are key regulators of glycolysis because they control the intracellular level of fructose-2,6-bisphosphate, a potent allosteric activator of phosphofructokinase-1 and thus an important determinant of glycolytic rate [26]. Previous work has shown that acetylation-induced cytoplasmic accumulation of PFKFB3 promotes glycolysis and protects cells against cisplatin-induced apoptosis [27]. In this context, the elevated expression of *pfkfb4a* observed here is consistent with a glycolysis-related transcriptional response. Although glycolytic flux was not directly measured, the upregulation of *pfkfb4a* suggests that *dnmt3b* knockdown may be associated with altered expression of glycolysis-related genes under high-glucose stress. Therefore, the transcriptional changes induced by *dnmt3b* knockdown appear to involve both glucose transporter-related and glycolysis-related genes.

Another notable finding was the significant reduction in *il6* and *nfκb* expression following *dnmt3b* knockdown. NFκB is widely recognized as a central hub linking metabolic disturbance with inflammatory signaling, and its activation can aggravate tissue injury through reciprocal interactions with altered metabolic programs [28]. Our data suggest that *dnmt3b* knockdown was associated with reduced expression of inflammatory markers under high-glucose stress. This interpretation is supported by previous evidence showing that DNMT3B can influence TNF Receptor-Associated Factor 2 (TRAF2)-mediated NFκB pathway activation through methylation-dependent regulation, thereby enhancing inflammatory signaling [29]. In addition, Lian et al. demonstrated that IL6 expression is highly sensitive to CpG methylation at specific loci, and that methylation changes at even a single CpG site can significantly alter IL6 transcription [30]. These findings are in line with the anti-inflammatory trend observed in our study. AOC1 encodes amine oxidase copper-containing 1, an enzyme closely associated with DAO activity and bioactive amine metabolism. DAO has been reported as an indicator associated with intestinal mucosal maturation, integrity, and damage-repair status [31]. In the present study, *aoc1* expression increased after *dnmt3b* knockdown, whereas it did not show significant differences among 5MC treatment groups. Therefore, the *aoc1* result was interpreted as an epithelial status-related transcriptional response rather than direct evidence of improved intestinal barrier function. The increased *aoc*1 expression after *dnmt3b* knockdown may indicate an epithelial status-related transcriptional response associated with amine metabolism. Taken together, *dnmt3b* knockdown was associated with coordinated transcriptional changes in glucose transporter-related genes, glycolysis-related genes, epithelial status-related genes, and inflammatory markers. However, because DNA methylation levels, direct glucose uptake, and rescue experiments were not assessed, these findings should be interpreted as DNMT3B-associated transcriptional responses rather than evidence for a confirmed methylation-dependent regulatory mechanism.

### 3.3. Exogenous 5MC Alleviates High-Glucose-Induced Metabolic and Epithelial Stress Associated with dnmt3b–cdx1b–sglt1-Related Transcriptional Responses

In fish, digestible carbohydrates are rapidly hydrolyzed by digestive enzymes in the intestine and subsequently transported into the body. When carbohydrate intake or absorption rate exceeds the metabolic capacity of the organism, postprandial hyperglycemia or sustained hyperglycemia may occur [32]. Chronic hyperglycemia not only disrupts endocrine and energy metabolic homeostasis, but may also induce abnormal lipid deposition, intestinal barrier impairment, and aggravated inflammatory responses, thereby ultimately suppressing growth [33]. In the present high-glucose culture system, supplementation with 6 mM 5MC significantly reduced glucose levels in the culture medium, while simultaneously decreasing LDH and DAO activities as well as MDA content. Because extracellular glucose levels may be affected by cellular viability and metabolic status, this result is interpreted as a reduction in residual glucose in the medium rather than direct proof of enhanced glucose uptake. These findings suggest that 5MC may reduce extracellular stress- and injury-associated indicators under high-glucose exposure. Cell viability analysis further showed that 6 mM 5MC improved cell survival under high-glucose stress, whereas this beneficial effect weakened at higher concentrations. In particular, the marked increase in LDH activity at 24 mM suggests that excessive 5MC may induce cytotoxicity or membrane injury. Thus, 6 mM appears to represent a relatively favorable concentration in this in vitro model, while 24 mM may exceed the safe range of grass carp intestinal epithelial cells. The different PK activity trends observed after 48 h of 5MC treatment and after an additional 12 h of reculture may reflect stage-dependent cellular responses. PK catalyzes the final ATP-generating step of glycolysis and is closely related to cellular energy metabolism [34]. However, because PK is mainly an intracellular enzyme, its activity in the culture supernatant may also be affected by membrane integrity and enzyme leakage, as indicated by LDH-based cytotoxicity assays [35]. It should be noted that extracellular glucose, LDH, DAO, and PK activities were measured in the culture supernatant and therefore do not directly represent intracellular glucose uptake, glycolytic flux, or intracellular enzyme activity. The reduction in extracellular glucose reflects decreased residual glucose in the medium, while LDH activity mainly indicates cell injury or membrane integrity-related changes. Moreover, because PK is primarily an intracellular glycolytic enzyme, its activity in the culture supernatant may be influenced by both glycolytic adaptation and enzyme leakage caused by changes in membrane integrity. Thus, the inconsistent PK trends between the 48 h treatment and the additional 12 h reculture more likely reflect stage-dependent extracellular stress responses rather than a direct linear change in intracellular glycolytic activity.

This interpretation is consistent with previous studies in fish showing that high-carbohydrate diets are commonly accompanied by elevated markers of intestinal injury. For example, plasma DAO levels increase significantly with increasing dietary starch levels in largemouth bass [36]. Xie et al. further reported that plasma LDH activity was significantly higher in the high-starch group than in the control group [37], and similar observations have also been reported in grass carp [38]. In Nile tilapia (*Oreochromis niloticus*) and pikeperch (*Sander lucioperca*), high-carbohydrate diets have been shown to reduce hepatic GSH content while increasing MDA levels [39,40]. In the present study, 6 mM 5MC supplementation for 48 h increased intracellular GSH content and decreased MDA levels, and this effect remained detectable after an additional 12 h of reculture, suggesting that the antioxidant-related changes induced by 5MC remained detectable after short-term reculture under high-glucose conditions. Together with the improved cell viability observed at 6 mM, these antioxidant changes suggest that 6 mM 5MC was associated with improved cellular status under high-glucose stress.

Several methyl donor-related or one-carbon metabolism-related nutritional factors have been reported to show similar protective effects. Betaine, as a methyl donor, can reduce high-carbohydrate-induced MDA accumulation and enhance antioxidant responses, thereby alleviating oxidative stress [41]. Likewise, sodium butyrate, a short-chain fatty acid and well-recognized Histone Deacetylase (HDAC) inhibitor, has been shown in fish feeding trials to reduce hepatic MDA levels and improve overall antioxidant capacity [42]. In addition, interventions with sodium butyrate or *Clostridium butyricum* can reduce permeability-related indicators such as serum DAO, suggesting that these nutritional factors may influence oxidative stress- and intestinal injury-related responses through multiple mechanisms [43]. Evidence from mammalian and cell models points in a similar direction. Meta-analysis of folate supplementation has shown that folate can reduce MDA levels while increasing GSH content, indicating a stable association between methyl donor availability and enhanced antioxidant defense [44]. In cell injury models, folate or its active form 5-Methyltetrahydrofolate (5-MTHF) has been shown to reduce LDH release and improve antioxidant indices including GSH [45,46]. Moreover, exogenous supplementation with SAM, a direct methyl donor, has also been reported to reduce tissue MDA levels in diabetes-related animal models [47]. In the present study, SAM levels were significantly lower in the NC and 6 mM groups than in the HG, 12 mM, and 24 mM groups. Because SAM is the methyl donor used by DNA methyltransferases during cytosine methylation, changes in SAM levels may reflect alterations in one-carbon/methyl donor-related metabolic status. However, SAM concentration alone cannot determine whether DNA methylation was increased or decreased. Therefore, the SAM result was interpreted only as an auxiliary metabolic indicator and was not used as evidence for a DNA methylation-dependent mechanism. Taking together, these findings suggest that exogenous 5MC was associated with reduced oxidative damage- and cell injury-related indicators under high-glucose stress. Notably, the 6 mM 5MC group also exhibited a significantly increased cell migration rate, further suggesting that 5MC was associated with an increased wound healing rate under the present in vitro conditions [48]. This result is consistent with the observed changes in LDH, MDA, GSH, SAM and cell viability, and supports the view that 5MC was associated with improved cellular status in this high-glucose in vitro model.

It is worth noting that DNA 5-methylcytosine is widely regarded as a repressive epigenetic mark in many genomic contexts associated with transcriptional repression [49]. However, exogenous 5MC is a modified DNA base rather than a canonical metabolically active methyl donor; therefore, its biological effects should not be directly equated with DNA methylation regulation unless cellular uptake, DNA incorporation, and methylation changes are verified. In the present study, exogenous supplementation with 6 mM 5MC suppressed *dnmt3b* expression, while activating the expression of *cdx1b*, *sglt1*, *pfkfb4a*, and *slc2a3a*, all of which are closely related to intestinal nutrient transport and glucose metabolism. Importantly, the direction of these transcriptional changes was highly consistent with that observed in the *dnmt3b* knockdown experiment. This consistency suggests that the effects of 5MC and *dnmt3b* knockdown shared similar transcriptional features. However, the present data do not demonstrate that 5MC acts through *dnmt3b*-dependent regulation. It should also be emphasized that the present study did not determine whether exogenous 5MC was taken up by grass carp intestinal epithelial cells, metabolically utilized, or incorporated into genomic DNA. Thus, the observed cellular responses cannot be directly attributed to 5MC-derived DNA methylation changes. Future studies should combine labeled 5MC tracing, intracellular metabolite profiling, and locus-specific or genome-wide DNA methylation analyses to clarify the metabolic fate and possible methylation-related consequences of exogenous 5MC.

In addition, although cross-reactivity information for the mammalian primary antibodies is provided in Table S1, the use of non-fish-specific antibodies, particularly the CDX2 antibody used to detect a CDX2/CDX1B-like immunoreactive signal, remains a methodological limitation. Future studies should use fish-specific CDX1B antibodies or targeted proteomic approaches to further confirm the protein-level changes of CDX1B in grass carp intestinal epithelial cells. Second, the 6 mM 5MC concentration identified as effective in vitro cannot be directly translated into a dietary inclusion rate for live grass carp, and further in vivo studies are needed to determine its intestinal availability, metabolic fate, tissue distribution, dose–response relationship, and long-term safety. Third, although cell viability was assessed, apoptosis, proliferation, pH, and osmolality analyses were not included, limiting the evaluation of potential cytotoxic, osmotic, or proliferation-related effects at high 5MC concentrations. Finally, direct glucose uptake assays and targeted methylation analyses are still required to clarify whether and how 5MC is involved in glucose metabolism-related responses.

## 4. Materials and Methods

### 4.1. Cell Culture and Preparation of Complete Medium

Grass carp intestinal epithelial cells were obtained from the Shanghai Cell Bank. For cell recovery, cryovials were removed from liquid nitrogen and immediately thawed in a 37 °C water bath with gentle agitation. After thawing, the cell suspension was transferred into a centrifuge tube and mixed with 5 mL of high-glucose Dulbecco’s Modified Eagle Medium (DMEM, the basal glucose concentration was 25 mM) complete medium. The cells were centrifuged at 1000× *g* for 10 min, after which the supernatant was discarded, and the cell pellet was gently resuspended. The resuspended cells were then seeded into T25 culture flasks containing 5 mL of high-glucose DMEM complete medium and maintained at 37 °C in a humidified incubator with 5% CO_2_ and 95% relative humidity. Culture conditions were carried out according to the instructions provided by the Shanghai Cell Bank (Shanghai, China).

The high-glucose DMEM complete medium consisted of high-glucose DMEM (Gibco, New York, NY, USA), 1.0% penicillin-streptomycin solution (100×; Solarbio, Beijing, China), and 10% fetal bovine serum (Gibco, New York, NY, USA).

### 4.2. Experimental Design and Analytical Methods

#### 4.2.1. Evaluation of the Effects of *dnmt3b* Knockdown on High-Glucose Metabolism in Grass Carp Intestinal Epithelial Cells

##### Establishment of the High-Glucose Model

**Figure 11:**
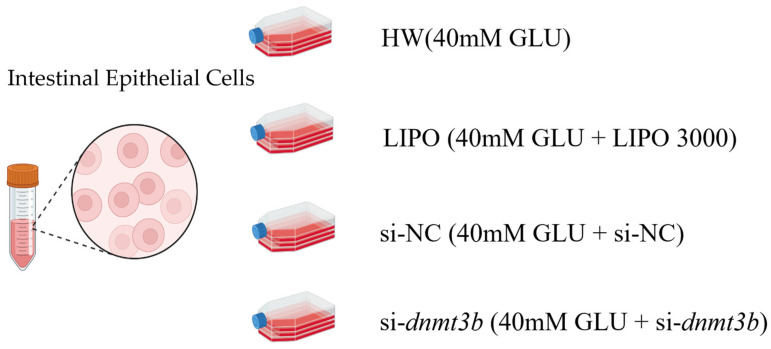
Evaluation of *dnmt3b* knockdown on high-glucose metabolism in grass carp intestinal epithelial cells.

Anhydrous glucose (≥98% purity, HPLC; 360.26 mg, Macklin, Shanghai, China) was added to the basal medium as 40 mM additional glucose, resulting in a final glucose concentration of 65 mM. The experiment consisted of four groups: the HW group, LIPO group, si-NC group, and si-*dnmt3b* group, with six replicates in each group. A schematic overview of the experimental procedure is presented in Figure 11.

##### Design and Synthesis of *dnmt3b* siRNA

The grass carp *dnmt3b* gene sequence (GenBank accession no. XM_051879002.1) was retrieved from GenBank, and the sequences of the Negative control (NC) and *dnmt3b*-siRNA were designed accordingly. The sequences are listed in Table 1. All oligonucleotides were synthesized by Shanghai Hanheng Biotechnology Co., Ltd. (Shanghai, China).

##### RT-qPCR and Western Blot Analyses

Cells were transfected with *dnmt3b* siRNA at a final concentration of 50 nM using Lipomaster 3000 Reagent (Vazyme, Nanjing, China) and harvested 48 h after transfection for RT-qPCR and Western blot analyses. When cell confluence reached 80–90%, the cells were harvested for real-time quantitative PCR (RT-qPCR) analysis. Briefly, the culture medium in the 6-well plates was removed, and the cells were washed three times with phosphate-buffered saline (PBS). After complete removal of the residual liquid, 500 µL of Trizol reagent (Takara, Dalian, China) was added to each well. The lysates were then transferred to 2 mL centrifuge tubes, and the cells were further homogenized using a homogenizer. Total RNA was extracted using an RNAiso Plus kit (Takara, Dalian, China). The quality and concentration of the extracted RNA were determined using a NanoDrop 2000 spectrophotometer (Thermo Fisher Scientific, Waltham, MA, USA). The genes analyzed in this study included *dnmt3b*, *cdx1b*, *sglt1*, *aoc1*, *il6*, *nfκb*, *pfkfb4a* and *slc2a3a*. *β-actin* and *ef1α* were used as the reference genes. Relative gene expression levels were calculated using the 2^−ΔΔCt^ method. The primer sequences used in this study are listed in Table 2.

Western blot analysis was performed according to the laboratory protocol described by Jiang [50]. Grass carp intestinal epithelial cells were lysed with RIPA buffer (Beyotime Biotechnology, Shanghai, China), and the protein concentration in the supernatant was adjusted to 2.5 mg/mL using the BCA protein assay. Protein samples were mixed with 5× loading buffer (Beyotime Biotechnology, Shanghai, China) and denatured prior to electrophoresis. The proteins were separated using an SDS-PAGE gel preparation kit and electrophoresis buffer (Servicebio, Wuhan, China) and then transferred onto PVDF membranes. The membranes were blocked with blocking buffer (Beyotime Biotechnology, Shanghai, China) at room temperature for 1 h and washed three times.

The PVDF membranes were incubated at 4 °C for 12 h with the following primary antibodies: DNMT3B (WL06255, 1:1000), CDX2/CDX1B-like (ET1605-4, 1:1500), SGLT1 (ER1916-51, 1:1500), PFKFB (ET1705-66, 1:750), NFκB (ET1603-12, 1:7500), and βactin (AF5003, 1:3000). The DNMT3B antibody was provided by Wanleibio (Shenyang, China). The CDX2/CDX1B-like, SGLT1, PFKFB, NFκB antibodies were obtained from HUABIO (Hangzhou, China). The βactin antibody was provided by Beyotime Biotechnology (Shanghai, China). To assess potential cross-reactivity, the immunogen or target regions of mammalian primary antibodies were aligned with the corresponding grass carp protein sequences, and amino acid identity/similarity and predicted molecular weight were summarized in Table S1. After washing three times, the membranes were incubated with horseradish peroxidase-conjugated goat anti-rabbit IgG (H+L) secondary antibody (Beyotime, A0208, 1:2000). Protein bands were visualized using an ultrasensitive ECL chemiluminescence kit (Beyotime, China). β-actin was used as internal control, and the gray values of each target protein band were quantified using ImageJ (V1.8.0.112) software.

#### 4.2.2. Evaluation of the Effects of Different Concentrations of 5-Methylcytosine on High-Glucose Metabolism in Grass Carp Intestinal Epithelial Cells

##### Experimental Design

A total of 150.16 mg of 5MC (≥ 98% purity, HPLC; Yuanye, Shanghai, China) was dissolved in 50.00 mL of sterile high-glucose medium containing 40 mM additional glucose (final glucose concentration, 65 mM), followed by sterilization through membrane filtration to obtain a 24 mM sterile 5MC stock solution. The stock solution was subsequently diluted with 40 mM high-glucose medium to prepare 5MC working solutions at final concentrations of 3, 6, and 12 mM. Six treatment groups were established: the control group (NC), high-glucose group (HG), and 5MC supplementation groups at 3, 6, 12, and 24 mM, designated as the 3 mM, 6 mM, 12 mM, and 24 mM groups, respectively. Each group contained three replicates.

**Figure 12:**
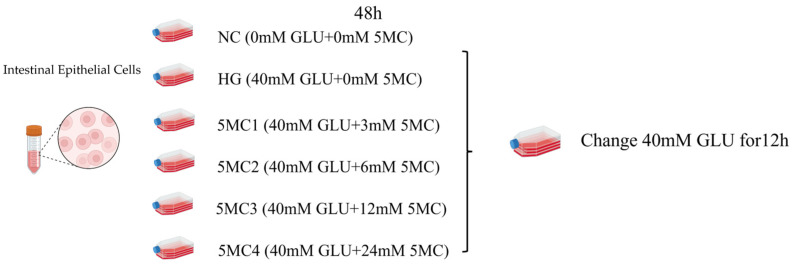
Evaluation of high glucose metabolism in intestinal epithelial cells of grass carp supplemented with different concentrations of 5-methylcytosine.

Different working solutions were mixed with an equal volume of cell suspension (6 × 10^6^ cells/mL) and seeded into 6-well culture plates. The cells were then exposed to the corresponding treatments at 37 °C in a humidified incubator containing 5% CO_2_ and 95% relative humidity for 48 h, until the cells had completely covered the well surface. Subsequently, the cells were digested with 0.05% trypsin (Gibco, New York, NY, USA), resuspended in 40 mM high-glucose medium, and re-seeded into 6-well plates, followed by incubation under the same conditions for an additional 12 h. A schematic overview of the experimental procedure is shown in Figure 12.

##### Biochemical Assays, RT-qPCR, and Western Blot Analyses

At the end of the 48 h treatment period and again after an additional 12 h of culture following replacement with high-glucose medium, the culture supernatants were collected for the determination of physiological and biochemical parameters. Glucose (GLU, Cat. No. A154-2-1), glutathione (GSH, Cat. No. A006-2-1), and malondialdehyde (MDA, Cat. No. A003-1-2) contents, as well as the activities of pyruvate kinase (PK, Cat. No. A076-1-2), hexokinase (HK, Cat. No. A077-4-1), lactate dehydrogenase (LDH, Cat. No. A020-2-2), and diamine oxidase (DAO, Cat. No. A088-3-1), were measured according to the manufacturers’ instructions using commercial assay kits (Nanjing Jiancheng Bioengineering Institute, Nanjing, China). The methods used for RT-qPCR and Western blot analyses were the same as those described in Section RT-qPCR and Western Blot Analyses.

### 4.3. Molecular Docking

The protein structures of Dnmt3b, Cdx1b, and Sglt1 were obtained from the UniProtKB database. The structure of Dnmt3b was separately submitted together with that of each target protein to the GRAMM online server for rigid-body protein–protein docking using the default parameters. After completion of the docking calculations, the top 10 candidate complex conformations ranked by the platform were retrieved. Binding energy was used as the primary evaluation criterion. In addition, the interaction interface area, hydrogen bonding, and key amino acid residues involved in the protein–protein interactions were comprehensively analyzed to identify the optimal docking conformation. After docking, binding free energy was calculated using PDBePISA (https://www.ebi.ac.uk/pdbe/pisa/, accessed on 21 May 2026), and the docking models were visualized with PyMOL 3.1.

### 4.4. Cell Viability

Cell viability of grass carp intestinal epithelial cells after treatment with different concentrations of 5MC was determined using a CCK-8 Cell Counting Kit (Vazyme, Nanjing, China). Cells in the logarithmic growth phase were harvested, digested, resuspended, and adjusted to an appropriate density. The cell suspension was then seeded into 96-well culture plates at 100 μL per well. After cell attachment, the NC group, HG group, and HG groups supplemented with different concentrations of 5MC were established according to the experimental design. Each group contained 12 replicate wells. Blank wells containing only culture medium and CCK-8 reagent without cells were also included.

After treatment, 10 μL of CCK-8 solution was added to each well and gently mixed. The plates were incubated in the dark at 37 °C in a humidified incubator containing 5% CO_2_ for 1–4 h. After incubation, the absorbance of each well was measured at 450 nm using a microplate reader. Cell viability was calculated using the following formula:

Cell viability (%) = [(OD treatment group − OD blank well)/(OD control group − OD blank well)] × 100%

where OD treatment group represents the absorbance value of the 5MC-treated groups at different concentrations, OD control group represents the absorbance value of the HG or NC group, and OD blank well represents the absorbance value of the cell-free blank wells. The experiment was repeated three times, and the results are expressed as the mean ± SEM.

### 4.5. SAM Content

SAM contents were determined using a commercial ELISA kit (Cat. No. ml035562) according to the manufacturer’s instructions (Mlbio, Shanghai, China). Briefly, adherent cells were gently washed with cold PBS and digested with 0.05% trypsin. Cells were collected by centrifugation at 1000× *g* for 5 min and washed three times with cold PBS. For cell lysis, 200 μL PBS was added to every 1 × 10^6^ cells, followed by repeated freeze–thaw cycles. The lysates were centrifuged at 1500× *g* for 10 min, and the supernatants were collected for SAM measurement.

For the ELISA assay, standard wells, blank wells, and sample wells were prepared. Standard wells were loaded with 50 μL of standards at different concentrations, while sample wells were loaded with 50 μL of cell extract. Except for the blank wells, 100 μL of horseradish peroxidase (HRP)-labeled detection antigen was added to each well. The plate was sealed and incubated at 37 °C for 60 min. After incubation, the liquid was discarded, and the plate was washed five times with washing buffer. Subsequently, 100 μL of substrate solution, prepared by mixing substrate A and substrate B at a 1:1 ratio, was added to each well and incubated at 37 °C for 15 min in the dark. Finally, 50 μL of stop solution was added to each well, and the absorbance was measured using a microplate reader.

A standard curve was generated using the standard concentrations as the *x*-axis and the corresponding optical density values as the *y*-axis. The SAM concentration in each sample was calculated using a four-parameter logistic regression model.

### 4.6. Wound Healing Assay

The wound healing assay was performed with reference to the procedure described by Gong [51]. Cells were seeded into 6-well plates and cultured until approximately 90% confluence. A linear scratch was then created using a sterile pipette tip. After washing with phosphate-buffered saline to remove detached cells, serum-free DMEM was added, and the cells were further cultured. Images were captured at fixed positions under a microscope at 24 h to record edge migration of the cells. Cell migration rate was quantified using ImageJ software. For each group, three images were selected, and three randomly chosen intervals were measured in each image to calculate the migration rate. The cell migration rate (wound healing rate) was calculated as follows:

Wound healing rate = (mean initial wound width − mean wound width at time t)/mean initial wound width

### 4.7. Statistical Analysis

All data were analyzed using SPSS 22.0 software (SPSS Inc., Chicago, IL, USA). After confirmation of normality by the Shapiro–Wilk test and homogeneity of variance by Levene’s test, one-way analysis of variance (ANOVA) was performed. Data are presented as the mean ± standard error (SEM), and differences were considered statistically significant at *p* < 0.05.

## 5. Conclusions

In summary, exogenous 5MC, particularly at 6 mM, alleviated high-glucose-induced metabolic and epithelial stress in grass carp intestinal epithelial cells. These findings provide preliminary in vitro evidence for the cellular effects of 5MC under high-glucose stress, while its uptake, metabolic fate, DNA incorporation, methylation-related consequences, and functional mechanisms require further investigation.

## Figures and Tables

**Table 1:** Sequence for dnmt3b-siRNA.

Primer	Sequence (5′-3′)
NC sense strand	UUCUCCGAACGUGUCACGUTT
NC antisense strand	ACGUGACACGUUCGGAGAATT
*dnmt3b*-si sense strand	GGAGUACAAGGAUAAUAAATT
*dnmt3b*-si antisense strand	UUUAUUAUCCUUGUACUCCTT

**Table 2:** Primer sequence for RT-qPCR.

Gene	Forward Primer (5′-3′)	Reverse Primer (5′-3′)	GeneBank No.	Product Length (bps)	Amplification Efficiency (%)
*dnmt3b*	ATGACTGGAGCATCCGTGTG	GGTAAACACGATGGGGCTCA	XM_051881116.1	75	109.20
*cdx1b*	AAACTCGTCAGTCGGTCAGC	TCTCTTTAGCACGCCGGTTC	XM_051878812.1	275	106.30
*sglt1*	ACCCATTGACGACAAACATC	TTCTTCACGCACCTCTTCTG	XM_051909110.1	139	101.60
*aoc1*	TTTTAATTGACCTCTCGCTCCCA	GTCGTGTTGTACTTCTCCTGAGT	XM_051886247.1	193	104.90
*il6*	CAGCAGAATGGGGGAGTTATC	CTCGCAGAGTCTTGACATCCTT	XM_051873835.1	134	104.40
*nfkb*	GAAGAAGGATGTGGGAGATG	TGTTGTCGTAGATGGGCTGAG	KJ526214	197	109.2
*pfkfb4a*	TTGCATTACGGGACCAGGAC	AAGGCAGTTCCTCTGCAGTC	XM_051912745.1	192	97.90
*slc2a3a*	TAACCGTGCTACCAGCCATC	CTTTGCGGGCCTTTTCTTCC	XM_051873574.1	105	101.70
*ef1α*	CGCCAGTGTTGCCTTCGT	CGCTCAATCTTCCATCCCTT	XM_051872454.1	99	102.10
*β-actin*	ACCAGCACGACCTTGCAGTG	CTGGGATGCATTCGGTTTGA	GU385744	174	98.80

## Data Availability

Data supporting the findings of this study are available in the article and Supplementary Materials. Further inquiries should be addressed to the corresponding author.

## Supplementary Materials

The following supporting information can be downloaded at: https://www.mdpi.com/article/10.3390/ijms27135732/s1.

## Abbreviations

The following abbreviations are used in this manuscript:

5MC	5-Methylcytosine
AOC1	Amine oxidase copper-containing 1
Cdx1b	Caudal type homeobox 1b
DAO	Diamine Oxidase
DNMTs	DNA methyltransferase
DMEM	Dulbecco’s Modified Eagle Medium
E13	Embryonic day 13
GLU	Glucose
GSH	Glutathione
HK	Hexokinase
IL6	Interleukin-6
LDH	Lactate Dehydrogenase
MDA	Malondialdehyde
NFκb	Nuclear Factor Kappa-B
PBS	Phosphate-buffered saline
PK	Pyruvate Kinase
SLC2A	Solute carrier family 2A
SLC5A	Solute carrier family 5A
SEM	Standard Error
