# Supplementary material for: High-Glucose-Induced Metabolic and Epithelial Stress in Grass Carp Intestinal Epithelial Cells Associated with Methylation-Related Transcriptional Responses
Source: Int J Mol Sci. 2026 Jun 25;27(13):5732. doi: 10.3390/ijms27135732 (PMC13362540; doi:10.3390/ijms27135732)
Supplement: Supplementary file 1 [file ijms-27-05732-s001.zip › ijms-4303171-supplementary.pdf]

Table.S1 Cross-species sequence alignment of antibody immunogens with grass carp target proteins.

Antibody	Host species	Immunogen/target region	Grass carp target protein	Identity / similarity	Predicted size
DNMT3B	Rabbit	Human DNMT3B aa 61-110 / 853	Grass carp Dnmt3b	59.34%	97 kDa
CDX2	Rabbit	Human CDX2 aa 20-60	Grass carp Cdx1b/Cdx-like	49.28%	34 kDa
SGLT1	Rabbit	Human SGLT1 401-500/665	Grass carp Sglt1	73.99%	73 kDa
PFKFB	Rabbit	Human PFKFB aa 260-520 / 520	Grass carp Pfkfb4a/Pfkfb-like	71.43%	60 kDa
NFκB	Rabbit	Human RELA aa 490-540	Grass carp Nfκb-like	40.69%	65 kDa
